# Survey for asymptomatic malaria cases in low transmission settings of Iran under elimination programme

**DOI:** 10.1186/1475-2875-11-126

**Published:** 2012-04-25

**Authors:** Samaneh Zoghi, Akram A Mehrizi, Ahmad Raeisi, Ali A Haghdoost, Habibollah Turki, Reza Safari, Asadallah Ahmadi Kahanali, Sedigheh Zakeri

**Affiliations:** 1 Malaria and Vector Research Group (MVRG), Biotechnology Research Center (BRC), Institut Pasteur Iran, Tehran, P.O. Box 1316943551, Iran; 2 National Programme Manager for Malaria Control, Ministry of Health and Medical Education, Hafez Ave., Jomhouri Cross, P.O. Box 1134845764, Tehran, Iran; 3 Research Center for Modeling in Health, Kerman University of Medical Science, Kerman, Iran; 4 Research Center for Infectious and Tropical diseases, Hormozgan University of Medical Sciences, Bandar Abbas, Iran; 5 Hormozgan University of Medical Sciences, Bandar Abbas, Hormozgan, Iran; 6 Jiroft University of Medical Science, Jiroft, Kerman

## Abstract

**Background:**

In malaria endemic areas, continuous exposure to *Plasmodium* parasites leads to asymptomatic carriers that provide a fundamental reservoir of parasites, contributing to the persistence of malaria transmission. Therefore, in the present investigation, the presence and prevalence of malaria asymptomatic cases were determined to evaluate the reservoir of infection in two malaria endemic areas with a previous history of malaria transmission in the south of Iran, Bashagard and Ghale-Ganj districts of Hormozgan and Kerman provinces, respectively, where malaria transmission has been drastically reduced in the recent years.

**Methods:**

The population samples (n=500 from each of the studied areas) were randomly collected from non-febrile, long-term residing, aged two to over 60years, during 20092010. Three identical surveys were carried out in both study areas and in each phase all the consent participants were interviewed and clinically examined. In all, three surveys to detect hidden parasite reservoirs (both *Plasmodium falciparum* and *Plasmodium vivax*), thick and thin blood smears and a highly sensitive nested-PCR were applied. In addition, the sero-prevalence survey for detecting malaria exposure was done by using a serological marker.

**Results:**

In this study, *P. vivax* and *P. falciparum* parasites were not detected by light microscopy and nested-PCR assay in all three surveys of samples. Antibody responses against *P. vivax* and *P. falciparum* were detected in 1 % and 0.2 % of the total examined individuals, respectively, in Bashagard district. Regarding to Ghale-Ganj district, about 0.9% of the individuals had IgG -specific antibody to *P. vivax* at the first and second surveys, but at the third survey 0.45% of the participants had positive antibody to *P. vivax* parasite. IgG -specific antibody to *P. falciparum* was detected in 0.2% of the participants at the first and follow-up surveys. The overall regional differences were not statistically significant (*P*>0.05).

**Conclusion:**

Taken together, the lack of asymptomatic carrier with the evidence of extremely low sero-positive to both *P. vivax* and *P. falciparum* among examined individuals supported the limited recent transmission in the studied areas and, therefore, these parts of Iran have potential to eliminate the disease in the next few years. However, continued follow up and action are still needed in both studied areas and also in their neighbouring province, Sistan and Baluchistan, which has the highest reported cases of malaria in Iran and also, has the largest border line with Afghanistan and Pakistan, with no elimination activities. This data will provide useful information for managing elimination activities in Iran.

## Background

Malaria remains an important cause of mortality and morbidity in many parts of the world and it could have adverse impact on the population, both from a health and a socio-economic attitudes. In malaria endemic areas, clinical manifestation of *Plasmodium* infection varies from asymptomatic to severe and fatal malaria. In high transmission areas, continuous exposures to *Plasmodium* parasites lead to partial immunity and consequently, create asymptomatic carriers in a given population [1]. In addition, asymptomatic cases provide a fundamental reservoir of parasites and they might become gametocyte carriers, contributing in the persistence of malaria transmission [2]. Therefore, the presence of asymptomatic cases is a big challenge for the management of the elimination programme in any malaria endemic area. In order to achieve a successful elimination, detection of all parasite carriers by active case detection and then treatment of all cases must be considered to interrupt the malaria transmission in endemic areas.

Asymptomatic malaria infections were frequently described in high and intermediate transmission areas including Ghana [3,4], Kenya [2], Senegal [5,6], Gabon [7,8], Nigeria [9,10], Uganda [11], Thailand [12], Burma [13] and Yemen [14]. However, in recent years, such cases have also been reported from low endemic areas such as Amazon region of Brazil and Peru [15-23], Colombia [24], Solomon Island [25] and Principe [26]. Notably, John and colleagues [27] reported that administration of different malaria control interventions reduced the asymptomatic malaria cases in an unstable malaria transmission area of Kenya and also in high transmission endemic area of Sri Lanka [28]. Since symptomless malaria consequences in the persistence of the parasite reservoirs and increases malaria transmission in human population, it can interfere with malaria elimination strategies. Therefore, to achieve successful elimination and finally eradication of malaria from the world, survey on the presences and the prevalence of asymptomatic cases in diverse malaria settings is recommended.

In Iran, a country located in the south-west of Asia, malaria was a major health problem with approximately 30-40% of the total mortality during 19211949 [29]. The National Malaria Eradication Programme was initiated in 1957 and remarkable achievements were obtained in most parts of the country. However, due to different obstructions in the south and south-east regions, malaria transmission was maintained with more than 90% of the total malaria cases in these areas. Therefore, in these particular areas the eradication programme was re-oriented to a control programme in 1980 [29]. Interestingly, afterward, disease burden has significantly reduced in the south and south-eastern parts of the country, due to successful interventions in controlling malaria. Consequently, since 2009, Iran, with 10 other countries entered the malaria pre-elimination programme with the technical support from the World Health Organization [30,31].

Simultaneously, indoor residual spraying, long-lasting impregnated bed nets (LLINs), active case detection and case management with the first-line recommended therapy for uncomplicated malaria, artemisinin combination therapy (ACT), combined with improved diagnostic capacities in all health facilities were all employed with a greater rate in comparison with the malaria control programme in Iran. In consequence, towards the reduction in transmission, based on the report by the Center for Diseases Management and Control (CDMC), the total malaria cases in Iran gradually dropped from 11,460 cases in 2008 to 6,122 in 2009 and as low as 3,031 cases in 2010 (Iranian CDMC, surveillance report, unpublished).

It should be noted that prior to applying an elimination strategy, the targeting countries need to occupy some constructions of active case detection for appropriate treatment of the asymptomatic parasite carriers to prevent major cause of sustained disease transmission. Therefore, it is prerequisite to detect parasite carriers that are undiagnosed by the light microscopy method or persist in population after anti-malarial treatment caused by drug resistance. To facilitate and complete the elimination efforts in Iran, this investigation was designed to assess the presence of the parasite carriers free of clinical symptoms in two malaria endemic districts with a history of malaria transmission in the south of Iran, Bashagard and Ghale-Ganj districts of Hormozgan and Kerman provinces, where transmission has been drastically reduced in recent years. The results of this study demonstrate the effectiveness and feasibility of implementing malaria elimination interventions in these malaria endemic areas of Iran.

## Methods

### Study area

**Figure 1:**
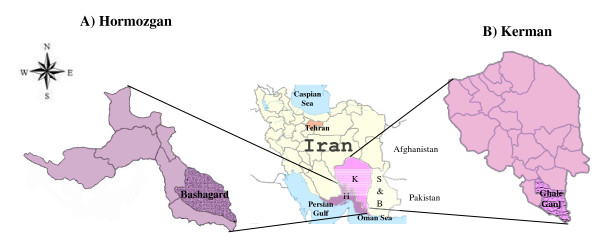
**Iran map showing the location of studied areas**. **A**) Bashagard district from Hormozgan province, **B**) Ghale-Ganj district from Kerman province in southern part of Iran**.** K=Kerman, H=Hormozgan, S & B=Sistan and Baluchistan.

This study was conducted in two malaria endemic areas with a previous history of malaria transmission in the south of Iran during 20092010. The transmission intensity in both study areas may also vary depending on the rainfall. The first study area was Bashagard district in the south-eastern part of Hormozgan province (Figure 1A) with a population of approximately 31,000 people. Bashagard is a tropical, mountainous area with a low level of population movement. The annual average temperature of Bashagard is 26C (7.7-44.2C) and the rainy season is from May to September with an annual pluviometer index of 11.6mm and relative humidity of 46.2%. According to the report of the CDMC in Iran, the annual parasite incidence [API=(confirmed cases during 1year/population under surveillance) x 1,000] in the Bashagard district was 0.08 in 2009 (Iranian CDMC, 2009 unpublished data). In this area, malaria is seasonal and transmission is year-round with two peaks, the first in June-July and the second in September-October and *P. vivax* parasite is the predominant species. The main malaria vector is *Anopheles stephensi*, which accounts for more than 90% of the anopheline fauna in Bashagard district. Different malaria control measures have been used in this region for the ultimate goal of the elimination since 2009. Therefore, the total cases of malaria in Bashagard district declined notably from 1,154 cases in 2008 to 197 and 11 cases in 2009 and 2010, respectively.

The second study area was Ghale-Ganj district in Kerman province in the southern part of Iran with a population of approximately 70,000 people (Figure 1B) and an annual average temperature of 26C. The API in the studied area was 0.08 in 2009 (CDMC 2009, unpublished data). In addition, malaria is seasonal and transmission is year-round with two peaks, the first from June-July and the second in October with *P. vivax* as the predominant species in this area and the main malaria vector is *An. stephensi*. Since 2009, the malaria elimination programme has been launched in this region; as a result, the total cases in Ghale-Ganj declined from 198 cases in 2008 to 62 and 13 cases in 2009 and 2010, respectively. It should be highlighted that comparable control measures were implemented in the both studied areas.

### Sample size calculation and sampling

This is the first study on detection of asymptomatic cases in two southern endemic areas, thus there is no available data for prevalence of asymptomatic cases either from other endemic region in Iran or from its neighbouring countries with comparable epidemiology. Assuming 3% as the minimum prevalence of asymptomatic malaria, the required sample size was 776 to estimate the prevalence with 0.9% (30% of the prevalence) with 95% confidence coefficient. In order to take the missing data into account, the sample size was increased by 20%.

Samplings were carried out after repeated meetings and discussions about the objectives of the project and its protocols with communities in both study districts. The population samples were collected from randomly long-term residing individuals between the ages of two and over 60years from Bashagard (n=500) and Ghale-Ganj (n=500) districts. The purpose of selecting different age groups was to detect hidden parasites within the high risk groups in different times of year (before, within and after local malaria transmission peaks) and samples were collected from August 2009 to March 2010 (Table 1) over two weeks by the regional malaria supervisor of each district.

**Table 1:** Blood sampling periods through this study in the southern malaria endemic areas of Iran

**Sampling periods in the study**	Bashagard*		Ghale-Ganj**	
	**No. of collected samples**	**Median age**	**No. of collected samples**	**Median age**
August 2009 (after the first peak)	500	20	500	18
October 2009 (within the second peak)	474	20	453	18
March 2010 (after the second peak)	456	19	442	18

*Malaria transmission in Bashagard district in Hormozgan province is year-round with two peaks, the first in June-July and the second in September-October.

**Malaria transmission in Ghale-Ganj district in Kerman province is year-round with two peaks, the first from June-July and the second in October.

Overall, three identical surveys were carried out in both study areas to check the variation of the prevalence in different malaria transmission peaks. In each phase all the consent participants were interviewed and clinically examined. In addition, for each individuals demographic data with the episode of malaria in the past 10years, anti-malarial treatment, and travels to the highly malaria endemic areas in neighbouring countries, Afghanistan and Pakistan, were recorded by questionnaire. Besides, exclusion criteria in this investigation were: non-consenting individuals during the follow-up study, symptomless individuals who preferred to be treated during follow-up, those individuals with a history of fever within the past 48hours ( 37.5C), having other malaria symptoms, patients who had received anti-malarial therapy in the past four weeks prior to the sampling and also during follow-up, pregnant women as well as children under two years old. During the second and third cross sectional surveys in both studied areas, some of the consent participants were lost due to death, absence in the area during the sampling period, pregnancy, travel to highly malaria endemic areas of Afghanistan and Pakistan, refusing to give blood and so on (Table 1).

Regarding the follow-up protocol for detection of asymptomatic carriers, in case of any microscopy and/or nested PCR positive individual (who remained symptomless for 60days, regardless of the onset of the infection), they were invited to go through the 60-day follow-up under the constant supervision of the local healthcares. They also were clinically examined and asked about malaria symptoms and at the same time blood smear was collected. All individuals who presented symptoms during the follow-up period were immediately treated and recorded as symptomatic patients, then excluded from the study.

In all three surveys and also the follow-up study to detect *Plasmodium* infection, firstly, thick and thin blood smears were prepared from all of the participants monthly by a well-trained microscopist in the local malaria health centres and were also cross-checked in Malaria and Vector Research Group (MVRG), Institut Pasteur, Iran. The initial slides were considered negative if no parasites were observed after examining 200 fields of Giemsa-stained thick blood smears or counting 1000 red blood cells (RBCs) in a Giemsa stained thin blood film at X1,000 magnification under immersion oil. Secondly, 2ml venous blood was collected in tubes containing EDTA for detection of sub-patent *Plasmodium* species by nested-PCR [32] and sero-positive individuals by serological marker.

In this work, informed consent was obtained before blood collection from adults or parents or legal guardians of children who were a participant in this study. This study was approved by the Ethical Review Committee of Research in Institut Pasteur, Iran.

### Assessment of *plasmodium vivax* and *plasmodium falciparum* prevalence by nested-PCR

DNA was extracted from 300L blood samples by using the commercially available DNA purification kit (Promega, Madison, WI, USA), and kept at 20C until use. DNA of *P. vivax* and *P. falciparum* was detected by nested-PCR amplification of the small sub-unit ribosomal ribonucleic acid (18ssrRNA) genes. The nest-1 PCR was performed similarly for both *Pasmodium* species using primers and cycling parameters described previously [32]. In addition, to detect *P. vivax* DNA, nest-2 PCR amplification was done by using newly designed forward primer (5-AGGACTTTCTTTGCTTCGGC-3) and reverse primer (5-AAGGCACTGAAGGAAGCAATC-3) in the MVRG lab to amplify a 419bp fragment. Amplification was carried out in a final volume of 25L including 1L nest-1 PCR product as a template, 250nM of primers, 10mM TrisHCl (pH 8.3), 50mM KCl, 2mM MgCl_2_, 125M of each of the four deoxynucleotide triphosphates and 0.4U of Taq polymerase (Invitrogen, Carlsbad, CA). The cycling condition for nest-2 PCR of *P. vivax* corresponding gene was as follows: 95C for 5min, 25 cycles of 94C for 1min, 58C for 1min, 72C for 1min followed by 72C for 15min. The nest-2 PCR amplification of *P. falciparum* was carried out as described previously [32]. Two negative controls were included in each set of amplification reactions, one with no DNA and the other was genomic DNA prepared from healthy individuals with no history of malaria, living in non-malaria endemic regions. In addition, extracted DNA from continuous culture of *P. falciparum* (K1 strain) and microscopically positive *P. vivax* were used as positive controls for the amplification of *P. falciparum* and *P. vivax* 18ssrRNA gene, respectively. All the products were visualized in 2% agarose gels containing ethidium bromide under ultraviolet transillumination.

### Production of recombinant PvMSP-1_19_ and PfMSP-1_19_ as antigens

In this study, the 19-kDaC-terminal region of merozoite surface protein 1 of *P. vivax* and *P. falciparum* (PvMSP-1_19_ and PfMSP-1_19_), one of the highly immunogenic antigen during the natural *Plasmodium* infection in human [33,34], was expressed and used as the recombinant antigen as described previously [35,36]. Briefly, the PvMSP-1_19_ (GenBank accession no. AY925098) was expressed as a recombinant protein fused to the C-terminal of poly-histidine (6-His) tag in *Escherichia coli* M15 and purified using Ni-NTA agarose (Qiagen, Germany) [35]. In addition, four pre-determined PfMSP-1_19_ variants in local *P. falciparum* isolates (E/TSR/L, E/TSG/L, E/KNG/F and Q/KNG/L with GenBank accession nos. HM569746, HM569747, HM569748 and HM569750 respectively) [36] were expressed as recombinant proteins fused to the C-terminus of glutathione S-transferase (GST) of *Schistosoma japonicum* using the pGEX-KG vector in *Escherichia coli* BL21 and purified by using glutathione sepharose 4B resin (Amersham Biosciences, USA) [36]. The fractions containing PfMSP-1_19_ were desalted with Econo-Pac 10DG columns (BioRad, USA) according to the manufacturers manual and analysed by SDS-PAGE on 12% gels. Afterward, immuno-blotting was performed to confirm the specificity of the expressed PvMSP-1_19_ with anti-His antibody (Qiagen, Germany, only for rPvMSP-1_19_) as well as positive *P. vivax* infected human sera and in case of rPfMSP-1_19_ only sera from positive infected human sera with this parasite were used.

### Detection of anti-PvMSP-1_19_ and -PfMSP-1_19_ specific antibodies using ELISA

Collected plasma samples from individuals of both study areas were used for the presence of the anti-PvMSP-1_19_- and -PfMSP-1_19_-specific IgG, IgG1 and IgG3 antibodies by indirect ELISAs as described previously with some minor modifications [35,36]. Briefly, Maxisorp flat-bottom, 96-well microplates (Grainer, LaborTechnic, Germany) were coated with 35 and 200ng (50ng of the each PfMSP-1_19_variant) of purified PvMSP-1_19_ and PfMSP-1_19_ antigens, respectively, in 0.06M carbonate-bicarbonate buffer (pH 9.6) and incubated at 4C overnight. After washing three times with PBS containing 0.05% Tween 20 (PBS-T), the microplates were blocked with 100l PBS containing 2% BSA and 0.05% Tween 20 (pH 7.4) at room temperature for 1h. In the next step, the sera samples were added to each well at 1:500 and 1:200 dilutions for PvMSP-1_19_ and PfMSP-1_19_ antigens, respectively. The plates were washed and incubated with 100l of anti-human IgG horseradish peroxidase (diluted 1:25,000 in PBS-T, Sigma, USA) for 1h at RT. Then, plates were washed and the enzyme reaction was developed with an enzyme-specific substrate, o-phenylenediamine dihydrochloride-H_2_O_2_ (OPD, Sigma, USA). The reaction was stopped by 2N sulfuric acid and the absorbance was measured at 490nm. To detect IgG1 and IgG3 subclasses, an ELISA was performed as described above except for the secondary antibodies that biotin-conjugated isotype-specific mouse anti-human IgGs (Sigma, USA) were used at dilution of 1:3,000 and after the washing step, streptavidin-peroxidase conjugate (Sigma, USA) was added at dilution of 1:2,500. The ELISA cut-offs were calculated from the mean of negative sera (n=30) of the healthy individuals non-exposed to malaria transmission plus three standard deviation (SD) for both recombinant antigens and the ODs more than cut-off values were considered as positive. In each plate, sera with patent *P. vivax* or *P. falciparum* infection were used as positive control.

### Avidity assay

Functional affinity of IgG-PvMSP-1_19_ or -PfMSP-1_19_ antibodies was determined in positive sera using avidity ELISA as described by Hedman and co-workers, with minor modifications [37]. Avidity index (AI) was calculated as the ratio of the OD of urea-treated wells to the OD value of untreated samples multiplied by 100. An AI less than 30% was considered low-avidity, between 30% and 50% as intermediate avidity and greater than 50% as high-avidity antibodies [37].

### Statistical analysis

The differences between the proportion of positive IgG responders in Bashagard and Ghale-Ganj districts were analysed using the Chi-square (*X*^*2*^) test. In addition, Wilcoxon signed rank test was used for comparison between the levels of the IgG antibody through various sampling stages. SPSS 16.0 for windows software (SPSS Inc. USA) was used for all statistical analysis. *P <*0.05 was considered significant.

## Results

### Demographic data

At the beginning of the first survey in both study districts a total of, 1,000 consent individuals (500 from each study area) accepted to participate in this study. However, in the two other surveys, during follow-up sampling, 456 (91.2%) individuals (median age, 19) from Bashagard and 442 (88.4%) individuals (median age, 18) from Ghale-Ganj districts had been recruitment and completed the second and third surveys (Table 1). In the present investigation, the distribution of the different age groups has been shown in Table 2, and the majority of the individuals were six to 15years old in both districts. About, 41% of the total individuals enrolled from both study areas had at least one microscopy-positive blood film in the past 10years and the majority of them had a history of infection with *P. vivax* parasite. This information was obtained based on filled questionnaires by consent participants and confirmed by the local healthcare recording documents.

**Table 2:** The distribution of age group among participants

	**Bashagard**	**Ghale-Ganj**
**Age group (y)**	**No. of samples (%)**	**No. of samples (%)**
**≤5**	10 (2)	11 (2.2)
**6-15**	160 (32)	196 (39.2)
**16-20**	96(19.2)	78 (15.6)
**21-40**	147 (29.4)	158 (31.6)
**>40**	87 (17.4)	57 (11.4)
**Total**	500 (100)	500 (100)

### *Plasmodium* infections prevalence using microscopy and nested-PCR methods

At the all-longitudinal active case detection in the three surveys in Bashagard and Ghale-Ganj districts, none of the examined blood subset collected through all three phases of follow-up were positive for *P. vivax* and/or *P. falciparum* parasites by using light microscopy and nested-PCR assays.

### Sero-prevalence of anti- MSP-1_19_ antibodies

The presence of anti-PvMSP-1_19_ and -PfMSP-1_19_ IgG were evaluated by ELISA using recombinant PvMSP-1_19_ and PfMSP-1_19_ as antigens. The level of OD_490nm_ and epidemiological data of the positive sera from Bashagard district at different phases of the study have been shown in Table 3A and 3B. In this area, 1% of the total examined individuals had positive IgG PvMSP-1_19_-specific antibody at the first, second and third phases of sampling in August (after the first local peak), October (within the second peak) 2009 and March (after the second peak) 2010 (Table 3A)(*P*>0.05). The mean level of IgG PvMSP-1_19_-specific antibody significantly decreased from 0.820.33 at the first survey to 0.640.1 in the second survey and finally to the 0.590.1 in the last survey (*P*<0.05, Wilcoxon signed rank test). Furthermore, 0.2% of the individuals in Bashagard district had IgG PfMSP-1_19_-specific antibody in the first survey (after the first peak) that was persistent in follow-up studies (Table 3B). All IgG PvMSP-1_19_- or PfMSP-1_19_-specific antibodies in positive sera had also positive IgG1 but not IgG3 subclasses antibodies to both recombinant antigens (Table 3)..

**Table 3:** **The antibody level (OD**_**490nm**_**) of anti-PvMSP-1**_**19**_**(A) -PfMSP-1**_**19**_**(B) IgG, IgG1 and IgG3 antibodies of sero-positive individuals in three surveys in Bashagard district, Hormozgan, Iran**

**A)**			Mean OD_490_*								
			**Survey 1**				**survey 2**			**Survey 3**	
**Code**	**Age (y)**	**sex**	**IgG**	**IgG1**	**IgG3**	**IgG**	**IgG1**	**IgG3**	**IgG**	**IgG1**	**IgG3**
**1**	12	M	0.61	0.52	-	0.55	0.51	-	0.48	0.20	-
**2**	15	F	0.77	0.42	-	0.76	0.41	-	0.73	0.41	-
**3**	18	M	1.40	1.04	-	0.72	0.44	-	0.6	0.35	-
**4**	21	F	0.55	0.40	-	0.52	0.35	-	0.48	0.30	-
**5**	28	F	0.77	0.40	-	0.68	0.37	-	0.65	0.32	-
**B)**			Mean OD_490_**								
			**Survey 1**				**survey 2**			**Survey 3**	
**Code**	**Age (y)**	**sex**	**IgG**	**IgG1**	**IgG3**	**IgG**	**IgG1**	**IgG3**	**IgG**	**IgG1**	**IgG3**
**1**	35	M	0.81	0.57	-	0.73	0.46	-	0.57	0.31	-

Regarding to Ghale-Ganj district, 0.9% of the individuals had IgG PvMSP-1_19_-specific antibody at the first and second surveys (Table 4A). Besides, at the third survey 0.45% of participants had positive IgG response to PvMSP-1_19_ antigen (Table 4A). The mean level of IgG PvMSP-1_19_-specific antibody decreased from 0.680.08 at the first to 0.590.08 in the second survey and finally to 0.520.1 in the third survey (*P>*0.05, Wilcoxon signed rank test). Regarding to IgG PfMSP-1_19_-specific antibody response, 0.2% of the participants showed positive antibody response at the first and follow-up surveys (Table 4B). Analysis of the subclasses antibodies showed IgG1 and IgG3 PvMSP-1_19_- or PfMSP-1_19_-specific antibodies in positive follow-up samples of Ghale-Ganj district (Table 4). In addition, comparison of the positive IgG responders in Bashagard and Ghale-Ganj districts did not show any significant difference (*P*>0.05).

**Table 4:** **The antibody level (OD**_**490nm**_**) of anti-PvMSP-1**_**19**_**(A) -PfMSP-1**_**19**_**(B) IgG, IgG1 and IgG3 antibodies in sero-positive individuals in three surveys in Ghale-Ganj district, Kerman province, Iran**

**A)**			Mean OD_490_*								
			**Survey 1**				**survey 2**			**Survey 3**	
**Code**	**Age(y)**	**sex**	**IgG**	**IgG1**	**IgG3**	**IgG**	**IgG1**	**IgG3**	**IgG**	**IgG1**	**IgG3**
**1**	17	F	0.58	0.57	-	0.49	0.3	-	0.43	0.19	-
**2**	17	M	0.65	0.79	-	0.57	0.61	-	0.55	0.58	-
**3**	18	M	0.78	0.8	-	0.61	0.42	-	0.45	0.2	-
**4**	60	M	0.72	0.47	0.95	0.69	0.41	0.86	0.67	0.4	0.8
**B)**			Mean OD_490_**								
			**Survey 1**				**survey 2**			**Survey 3**	
**Code**	**Age (y)**	**sex**	**IgG**	**IgG1**	**IgG3**	**IgG**	**IgG1**	**IgG3**	**IgG**	**IgG1**	**IgG3**
**1**	25	F	0.6	0.45	-	0.59	0.4	-	0.55	0.35	-

Additionally, the avidity index (AI) was measured for IgG-PvMSP-1_19_ and -PfMSP-1_19_ antibodies in positive sera. The results showed that in Bashagard district, the positive sera to PvMSP-1_19_ had low (60%) or intermediate (40%) IgG antibodies in the collected samples; however, the only positive serum to PfMSP-1_19_ antigen showed high avidity IgG antibody (Table 5). Concerning Ghale-Ganj district, 3 of 4 (75%) IgG positive sera for PvMSP-1_19_ had low and/or intermediate IgG antibodies in the analysed samples; however only 1 of 4 (25%) samples had high avidity IgG antibody. Regarding PfMSP-1_19_ antigen, the only positive sample had intermediate IgG antibodies (Table 5).

**Table 5:** **The distribution of anti-PvMSP-1**_**19**_**and -PfMSP-1**_**19**_**IgG antibodies avidity by age group in analysed individuals**

							**Avidity Index (AI)**	
	**Antigen**	**Code**	**Age**	**Age group (y)**	**sex**	**Survey 1**	**Survey 2**	**Survey 3**
**Bashagard district**	**PvMSP-1**_**19**_	**1**	**12**	6-14	**M**	26.75	29.54	29.43
		**2**	**15**	15-20	**F**	21.1	22.37	23.6
		**3**	**18**	15-20	**M**	33.57	33.67	49.83
		**4**	**21**	21-40	**F**	54.68	42.01	44.46
		**5**	**28**	21-40	**F**	20.3	19.57	20.5
	**PfMSP-1**_**19**_	**1**	**35**	21-40	**F**	55.84	57.58	59.77
**Ghale-Ganj district**	**PvMSP-1**_**19**_	**1**	**17**	15-20	**F**	35.64	36.27	21.59
		**2**	**17**	15-20	**M**	49.81	49	48.81
		**3**	**18**	15-20	**M**	24.83	24	23.5
		**4**	**60**	>40	**M**	77.14	75	74.7
	**PfMSP-1**_**19**_	**1**	**25**	21-40years	**F**	47.3	46.2	46

Low avidity IgG antibodies, Avidity Index (AI) <30%.

Intermediate avidity IgG antibodies, Avidity Index (AI) between 30 - 50%.

High avidity IgG antibodies, Avidity Index (AI) >50%.

## Discussion

The prevalence and incidence of malaria has been reducing as the result of passive and active case detection and also scaling up malaria surveillance and control interventions in Iran since 2008. In the present study, therefore, to achieve and maintain the malaria elimination campaign in Iran, the presence and prevalence of malaria asymptomatic cases as healthy carriers of parasites were determined to evaluate the reservoir of infections in two malaria endemic areas (Hormozgan and Kerman provinces) for the first time, by using light microscopy and nested-PCR methods.

According to the updated surveillance report in 2010 by the Iranian CDMC (unpublished), the total cases in Bashagard districts were 11 *P. vivax* mono-infection confirmed by microscopy method and all of them were indigenous. However, from Ghale-Ganj districts, 13 microscopically confirmed malaria cases were reported and of the 13 cases nine were indigenous while the rest of the cases were imported from Pakistan (n =1) and Afghanistan (n=3). Notably, only one indigenous malaria case from each study area was under four years old. Indeed, in concordance with Iranian CDMCs report, the present results confirmed and recommended that malaria incidence dramatically reduced throughout applying massive malaria control tools in these regions in recent years. Therefore, the absence of asymptomatic carries and the low incidence of malaria cases in the studied areas confirm that the achievement of malaria elimination can be easily obtained in the near future in both districts.

Undoubtedly, the individuals with very low density parasitaemia missed by microscopy represent a major challenge for a malaria surveillance system and it suggests the remaining potential for malaria transmission in a given populations. Therefore, to detect very low parasitaemia in the population under study, a highly sensitive nested-PCR method was used as it is a robust diagnostic tool to identify simultaneously *P. falciparum* and *P. vivax* parasite infections in epidemiological studies [38]. Remarkably, in this study, no hidden *Plasmodium* parasites were detected during all the three surveys. As a result, hidden parasite reservoirs are not a big challenge for the elimination campaign in these regions; however achieving the final phase of elimination and entering the eradication phase (malaria free) in these regions, active case detection of symptomatic and asymptomatic carries with the purpose of proper treatment should be continuously carried out. This data is also in agreement with the reported results from Sri Lanka [28] and Kenya [27] that confirmed reducing asymptomatic malaria in parallel to decreasing symptomatic malaria because of applying different malaria control interventions firmly. In contrast, the present results were against the reported findings from different malaria endemic regions in the world where the malaria endemicity is still considered as low transmission areas [16,19,23,24].

Consistent with the data from the Iranian CDMC (based on collected data from various health centres throughout the malaria endemic areas in the country) and using serological tools in this study, the results revealed that *P. vivax* was the most prevalent species in both study areas. Since this is the first sero-prevalence work using recombinant MSP-1_19_ in Iran, thus there is no possibility for direct comparison to previous studies. However, the explanation for the detection of a very low prevalence of anti-MSP-1_19_ among examined subjects in both studied areas might be explained by the extensive use of LLINs with other malaria interventions since 2008 that support the decline in the disease burden and thus, the absence of any re-infection.

Different studies reported that serological markers such as blood stage proteins of *Plasmodium* species (e.g. MSP-1_19_ and/or AMA-1) can be useful and reliable tools for evaluating the malaria transmission intensity, monitoring changes in malaria transmission as well as malaria control and elimination activities [39-41]. Indeed, the prevalence of the anti-MSP-1_19_ with an expected half-life of 150years [42] revealed the longer periods of exposure to malaria infection over many years and thus is correlated to the malaria transmission intensity. In the current investigation among all 1000 examined individuals there were no detectable *Plasmodium* species but an exposure of about <2% to antibodies against the merozoite stage of the parasites was recognized. As the overall sero-prevalence of anti-PvMSP-1_19_ and PfMSP-1_19_ was extremely low with no detectable asymptomatic infection by nested-PCR in the examined individuals and there were no clinical signs of malaria infections during the follow-up study, hence in the absence of the re-infection, anti-MSP-1_19_ antibodies could be sustained in the previous exposure to the parasites many years earlier. This result was also supported by the age of all sero-positive individuals that was above 10years. Also, the reduction of antibody level during the follow-up surveys was in accordance to previous studies showing that antibodies rising to PvMSP-1_19_ and PfMSP-1_19_ are short half-life in Brazil and Iran [33,36]. Furthermore, as shown by previous studies, high affinity/avidity antibodies are expected to play an important role in the effective humoral immune response and it has been associated with more repeated infections with increasing age [43,44]. In this investigation, with the absence of any evidence for re-infection, the avidity results did not change in any of the examined subjects during the follow-up period which might have required further avidity maturation.

Moreover, with the lack of hidden parasites from both study districts with the previous history of malaria transmission, the future surveillance and assessments of the hidden parasites should focus on areas with reported malaria cases in the past six-12months in these provinces. Also, as a critical part of malaria elimination is detecting the prevalence and the presence of hidden parasites in malaria endemic areas, thus, similar study is highly needed in the neighbouring province, Sistan and Baluchistan, with the highest reported cases of malaria in Iran. This province is located in the south-eastern part of Iran with the largest border line with Afghanistan and Pakistan as well as with both studied districts. Providing comparable data from Sistan and Baluchistan province with high rates of imported malaria cased from two highly endemic malaria countries, Afghanistan and Pakistan, with no elimination activities will reduce the chance of local epidemic and re-establishment of disease transmission.

## Conclusion

Taken together, the lack of asymptomatic carrier, a substantial decline in malaria cases and also tremendously low sero-positive of anti- PvMSP-1_19_ and PfMSP-1_19_ antibodies supported that the recent malaria elimination activities dropped the level of malaria in both studied districts. In addition, the results showed that these two studied districts in the south of Iran could successfully move from the pre-elimination phase toward eliminating the disease and it is achievable in the next few years. However, to avoid any problem during elimination activities in Iran, surveillance of symptomatic and asymptomatic malaria cases combined with other interventions including vector surveillance is very important in the implementation of malaria elimination in Iran. Therefore, to provide baseline data on the malaria situation in Iran toward designing malaria elimination and eradication strategies based on the local epidemiology, such survey could be extended to other areas at risk in particular, Sistan and Baluchistan province with the highest reported malaria cases in Iran.

## **Competing interests**

The authors declare that they have no competing interests.

## **Authors contributions**

SZ, AR and AAH designed the study. SZ developed the experimental protocols, supervised field and laboratory works, finalized the interpretation of the data and wrote down the manuscript. AR and AAH have also contributed to the analysis of the data and critically reading the content of the manuscript. SZ and AAM performed most of the laboratory work and also helped in analysing the data. HT helped partially in some of the laboratory work and also sampling in Bashagard district. RS and AA were also involved in field works and sampling in Bashagard and Ghale-Ganj districts, respectively. All authors read and approved the final manuscript.
